# Exceeding the recruitment target in a primary care paediatric trial: an evaluation of the Choice of Moisturiser for Eczema Treatment (COMET) feasibility randomised controlled trial

**DOI:** 10.1186/s13063-016-1659-8

**Published:** 2016-11-17

**Authors:** Kingsley Powell, Victoria J. Wilson, Niamh M. Redmond, Daisy M. Gaunt, Matthew J. Ridd

**Affiliations:** 1 Centre for Academic Primary Care, School of Social and Community Medicine, University of Bristol, 39 Whatley Road, Bristol, BS8 2PS UK; 2 National Institute for Health Research Collaborations for Leadership in Applied Health Research and Care West (NIHR CLAHRC West), University Hospitals Bristol NHS Foundation Trust, Bristol, UK; 3 Bristol Randomised Trials Collaboration, School of Social and Community Medicine, University of Bristol, 39 Whatley Road, Bristol, BS8 2PS UK

**Keywords:** Eczema, Recruitment, Randomised controlled trials, Children, Primary care

## Abstract

**Background:**

Recruiting to target in randomised controlled trials of investigational medicinal products (CTIMPs) in primary care and paediatric populations is notoriously difficult. More evidence is needed for effective recruitment strategies in these settings. We report on the impact of different recruitment strategies used in the Choice of Moisturiser in Eczema Treatment (COMET) study – a feasibility trial comparing the effectiveness of four emollients for the treatment of childhood eczema – recruiting via general practitioner (GP) surgeries.

**Methods:**

Initially, 16 GP practices invited potentially eligible children to take part in the trial by sending an invitation letter (self-referral pathway) or by consenting and randomising them into the study during a visit to the practice (in-consultation referral). Measures implemented during the study to maximise accrual included signing up six additional GP practices, increasing the upper age limit eligibility criterion from 3 to 5 years, and permitting healthcare professionals other than doctors to confirm participant eligibility. We used descriptive statistics and univariate linear regression models to explore associations with practice recruitment rates.

**Results:**

A total of 197 participants were recruited, exceeding the target of 160. Of these, 107 children entered via self-referral and 90 by in-consultation pathways. Of the recruited population, 12.6 % were aged between 3 and 5 years (the raised upper age limit). The six additional practices contributed 37.4 % (40 of 107) of participants recruited by self-referral. Only almost one-third (18 of 56 [32.1 %]) of potential recruiting clinicians recruited one or more participants in-consultation, which was a more problematic pathway because of data verification issues. Three research nurses and a pharmacist from four practices recruited 48.9 % (44 of 90) of participants via this pathway. Univariate linear regression models showed no evidence of association between the number of children recruited via the self-referral pathway by practice and practice list size (*p* = 0.092) or practice deprivation decile (*p* = 0.270), but practice deprivation was associated with a higher number of children recruited in-consultation (*p* = 0.020) by practice.

**Conclusions:**

Self-referral and in-consultation recruitment yielded similar numbers, but the in-consultation pathway was more problematic. Future trials of this type should consider the condition, normal care pathway and number of potentially eligible children and be prepared to use multiple recruitment strategies to achieve recruitment targets.

**Trial registration:**

ISRCTN21828118. Registered on 1 May 2014.

EudraCT2013-003001-26. Registered on 23 Dec 2013.

## Background

Successful recruitment is paramount for high-quality randomised controlled trials (RCTs). Failure to recruit to target can negatively affect the reliability of the trial results, costs and timely dissemination of the findings for clinical practice [1]. Because recruitment into trials is often challenging, identifying effective recruitment strategies is a common primary focus for trial methodology research because there is a lack of evidence about which strategies are most successful [2].

It is more challenging to recruit to clinical trials of investigational medicinal products (CTIMPs) compared with non-CTIMPs due to the potential higher risk for participants and the additional regulatory requirements. Recruitment problems faced by RCTs in primary care are also well documented [3–7]. Recruiting children has further complexities because of their vulnerable status, where parents’ primary concern is their child’s safety, meaning they might be more reluctant to consent to their child participating in an RCT [8]. Previous trials and qualitative studies in primary care suggest that good personal relationships with practice staff, financial incentives, simple recruitment criteria and referral processes, and support from a research nurse promote successful recruitment [5, 6, 9–17]. Paediatric trials have shown that parents may be more likely to allow their child to participate if the trial offers healthcare information, new treatments, enhanced care for their child, low burden of involvement and appeals to their altruistic nature [18, 19]. The role of the clinician, particularly the way in which the clinician communicates trial information, is also a key factor in recruiting children [8], but it is often hampered by poor clinician understanding of RCTs and difficulties with the informed consent process [20–22].

Two common methods of recruiting to primary care trials are (a) inviting patients by letter (mail-out) and (b) inviting patients seen in a consultation at the practice (opportunistic). For RCTs on long-term conditions, a mail-out is generally used, with opportunistic recruitment alongside if appropriate. For RCTs on acute conditions, the opportunistic method may be the only option. Very few paediatric trials in primary care have reported detailed data on these recruitment methods. Two non-CTIMP U.K. trials recruiting children with eczema from general practices by mail-out reported much lower response rates than expected, one of which had estimated a 50 % response rate, whereas in reality it was only 35 % [23, 24]. Large cohort studies of acute conditions (respiratory and urinary tract infections) have successfully recruited children in primary care through clinicians [25, 26], but the effectiveness of this recruitment method has not been reported in clinical trials.

The Choice of Moisturiser in Eczema Treatment (COMET) trial established the feasibility of a definitive trial comparing the clinical effectiveness and cost-effectiveness of emollients in the treatment of childhood eczema in primary care [27]. Classed as a CTIMP, by virtue of the nature of the intervention and the design of the trial, participants were recruited by both mail-out and opportunistic recruitment. In this report, our aim was to compare the effectiveness of two recruitment pathways and discuss additional recruitment strategies that enabled us to successfully recruit children with eczema into a prospective, randomised feasibility trial.

## Methods

### Trial design

The aims and methods of the trial have been comprehensively outlined in the protocol [28] and main results papers [27]. In summary, COMET was an RCT designed to establish the feasibility of recruiting and randomising young children with eczema in primary care to receive treatment with one of four commonly prescribed emollients, and following them for 3 months. The aim was to recruit 160 children aged between 1 month and 3 years old with doctor-diagnosed eczema and absence of a known sensitivity to the study emollients. A formal sample size was not required, owing to the feasibility nature of the trial. A target of 160 was chosen so that a true consent rate of 50 % (160 children participating, having invited 320 potentially eligible children) would be estimated with 95 % CI of the order 44–56 %.

Recruitment took place over a 10-month period (between July 2014 and April 2015) within general practitioner (GP) practices in the West of England. Participants entered the study by one of two pathways, described below: (1) self-referral or (2) in-consultation. Initially, 16 practices were invited to take part, recruiting via both pathways. Six additional practices were enrolled later, recruiting via the self-referral pathway only. Parents or carers of participants gave written informed consent. The study was approved by the National Research Ethics Service (NRES) Committee South West – Central Bristol (reference 13/SW/0297).

### Recruitment pathways

#### Self-referral

There were two ways in which a parent could self-refer their child: (1) responding to an invitation letter sent from their practice or (2) by responding to posters or flyers displayed in practice waiting rooms. For the former, practices identified children whose electronic medical records had an eczema Read-coded diagnosis: atopic dermatitis not otherwise specified (NOS), eczema NOS, infected eczema, atopic dermatitis/eczema, infantile eczema, flexural eczema or allergic eczema. GPs screened and excluded children at their discretion, providing a reason for the exclusion from among a pre-defined list or by choosing ‘other’ and explaining the decision. The remaining children were sent a letter inviting them to participate in the study, a participant information sheet, a reply slip with a personalised mail-out identification number (ID), and a pre-paid addressed envelope. The reply slip detailed whether the child wanted to take part in the study or the reason for declining participation. Posters and flyers were displayed in the waiting rooms of those practices that had agreed to participate in the trial.

Practices recorded the number of children screened; number eligible or reason for ineligibility; sex; mail-out ID; date of birth; and which children were included in the mail-out. The mail-outs were staggered, with the first eight practices sending invitations in the first three months of the study (June to August 2014), followed by the remaining eight practices between September and the end of November 2014. The latter practices also sent reminder letters three months after the initial mail-outs to all non-responders who still met the eligibility criteria. Responses were received directly by the study team via post, telephone or email. The study team contacted the child’s parent/carer to check their eligibility. This involved four eligibility screening questions: (1) age of child, (2) confirmation of doctor-diagnosed eczema, (3) adult with parental responsibility able to give consent and (4) no known sensitivity to any of the study emollients. The study team received a consent form from those eligible, randomised the child and asked the relevant practice to issue the emollient prescription.

Practices were paid NHS Service Support Costs (SSC), defined as clinician reimbursement costs for time spent supporting recruitment of £288.93 for the site initiation visit, initial search and mail-out, and £281.80 for the reminder letter and provision of rooms for those participants who preferred to be seen at the practice rather than at their home. Only two children opted to be seen at their practice.

#### In-consultation referral

Children were also invited to take part in the study during a GP consultation at their practice, or an ‘in-consultation referral’. Also termed *opportunistic*, this method involved practice staff (mainly GPs and nurses) identifying eligible children in their day-to-day consultations and inviting them to take part. A good clinical practice (GCP)-trained GP signed the enrolment form, confirming eligibility. A GCP-trained member of staff received consent and randomised the child, and the practice immediately issued the relevant prescription. The completed trial paperwork was faxed to the study team, and the baseline visit was arranged. Practices received SSC of £91.78 per child randomised.

### Practice ‘research level’

The recruiting practices were members of the West of England National Institute for Health Research Clinical Research Network (NIHR CRN), which operates a research initiative scheme. The scheme provides funding to support infrastructure within primary care organisations to enable them to become and continue to be research-active. Practices carrying out trial procedures can operate at three levels, with those working at higher levels expected to support a large number of and more complex studies: level 1 is for practices that have proven they can deliver primary care research to a high standard and therefore are in a position to support NIHR CRN portfolio studies; level 2 is for experienced practices able to deliver on multiple NIHR CRN portfolio studies and/or studies of higher complexity; sessional funding level is for experienced practices that can request funded research time for practice staff. The practices recruited into COMET varied in their research level, with eight operating at level 1, eight at level 2 and six at the sessional funding level.

### Recruitment monitoring

Recruitment was monitored on a monthly basis and compared with the recruitment target. The accrual target per month was based on the average number of participants per month over 10 months, and then adjusted to account for season and time point in the study. For example, the monthly targets were less at the start of the trial and for December because previous experience has shown that recruitment is slower at these times. Despite this, accrual for the first three months of the trial was 15 children, compared with a planned target of 32. Consequently, additional strategies, described below, were implemented to try to improve recruitment.

### Strategies to increase recruitment

The upper age limit was increased from 3 to 5 years of age: 3 years had originally been chosen because the majority of children are diagnosed by this age and we anticipated that families would be less likely to have an established routine with a particular emollient than older children. The requirement of doctor-diagnosed eczema was changed to allow a diagnosis by any ‘appropriately qualified health professional’: The protocol was written with the assumption that the majority of children were given their diagnosis by a doctor (usually their GP), but practices fed back at an early stage that nurse practitioners working in advanced roles often independently made the diagnosis, and therefore the eligibility criteria did not fit with the usual care pathway in primary care.

Permitting healthcare professionals other than doctors to confirm participant eligibility: Originally, only doctors were allowed to sign off eligibility, due to the GCP principle which states, ‘[T]he medical care given to, and medical decisions made on behalf of, subjects should always be the responsibility of a qualified physician’ [29], page 9. Because this was a low-risk study with very simple eligibility criteria, in agreement with the study sponsor and through discussion with the Medicines and Healthcare products Regulatory Agency, restrictions on who could receive consent (GCP-trained health professional) and who could confirm eligibility (GCP-trained GP) were relaxed to allow any appropriately qualified, GCP-trained health professional to confirm eligibility and any appropriately qualified health professional to receive consent, provided it was countersigned by a GCP-trained professional. This simplified the referral process for in-consultation referral and increased the number of practice staff who could be involved in recruitment.

Ultimately, we signed up six more practices to recruit by the self-referral pathway only, totalling 22 practices. This second wave of practices undertook a single mail-out (no reminder letter) in February and March 2015 which included children in the extended age bracket (3–5 years).

Alongside these strategies, we encouraged in-consultation recruitment in the first wave of practices by providing regular email support; regularly sending to practices newsletters which included a graph of recruitment per practice to generate competition between practices; awarding a prize for the top recruiting clinician; and re-visiting most (10 of 16) practices recruiting by in-consultation to remind them of the recruitment process and to encourage more clinician involvement.

### Data analysis

We evaluated the impact of these strategies using post hoc descriptive analysis using Excel (Microsoft, Redmond, WA, USA) and Stata 14.1 (StataCorp, College Station, TX, USA) software. We examined and report recruitment rates overall, and by the two different recruitment pathways (self-referral and in consultation), using percentages and summary statistics. Univariate linear regression analyses were used to determine any association between the number of children recruited by either the in-consultation or the self-referral referral pathway by practice (outcome) and practice list size, deprivation ‘decile’, and the practice ‘research level’ for the in-consultation pathway outcome only (covariate). Practice size was defined as the number of registered patients at the practice, data which are publicly available. We did not look for an association between the self-referral pathway and the practice ‘research level’, because there are no grounds for expecting such an association.

## Results

### Recruitment rates

**Fig. 1:**
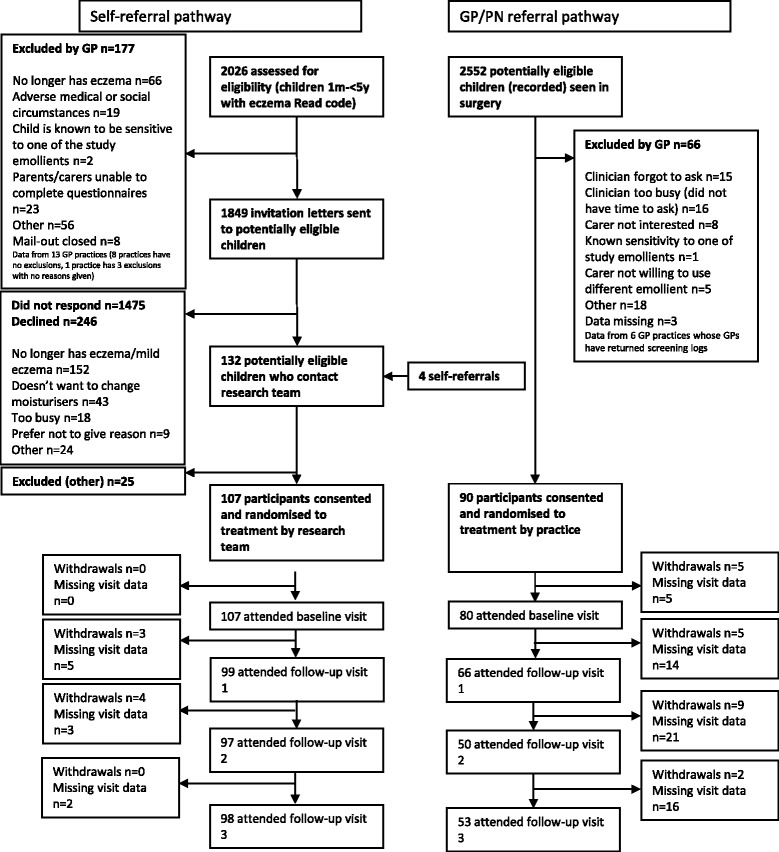
Consolidated Standards of Reporting Trials (CONSORT) diagram of recruitment by referral pathway. *GP* General practitioner, *PN* Practice nurse

The Consolidated Standards of Reporting Trials (CONSORT) [30] diagram in Fig. 1 shows recruitment by referral pathway. A total of 197 participants were recruited over 10 months, exceeding the original planned target of 160. Numbers of children recruited per pathway were similar, with 107 children entering the study by self-referral and 90 by in-consultation referral. The baseline characteristics of the children across the two pathways were generally similar. However, of the 14 % (28 of 197) who withdrew from the trial, most were recruited in-consultation (21 of 90 [23 %] versus 7 of 107 [7 %] of self-referrals). Retention is discussed more fully in the main feasibility paper [27].

**Fig. 2:**
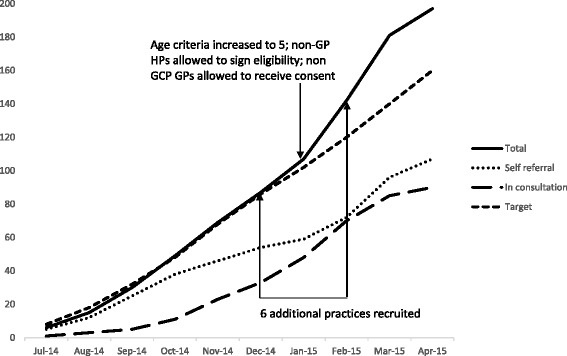
Cumulative recruitment in total and by referral pathway versus target recruitment and time points when key recruitment strategies were implemented. *GCP* Good clinical practice, *GP* General practitioner, *HP* Health professional

The cumulative recruitment by referral pathway and overall recruitment compared with recruitment target, as well as time points at which strategies to maximise recruitment were implemented, are shown in Fig. 2. We requested ethical approval for these new strategies in September 2014, when cumulative recruitment was well below target recruitment, but we did not receive approval until December 2014, when cumulative recruitment had improved. Figure 2 clearly shows the self-referral pathway as the more effective recruitment route in the first half of the recruitment period. The ‘dip’ seen in the recruitment by self-referral in January/February was due to the delay in recruiting additional practices and undertaking the mail-outs. There is a notable increase in the cumulative recruitment gradient for overall recruitment and recruitment by pathway following these strategies. Their impact is discussed in relation to the individual referral pathways.

### Recruitment by self-referral

A total of 2026 children were screened for eligibility from the 22 participating practices, and 1849 were sent an invitation letter in the self-referral pathway. Of these, 132 patients responded positively, of whom 25 were excluded and 107 were recruited. The main reasons for exclusion by the GP at the screening stage are listed in Table 1. The majority of invitees who responded to the invitation letter but declined to take part also gave ‘my child no longer has eczema’ as the reason (152 of 246 [61.8 %]). Non-responders from the original 16 practices, who still met the age eligibility criteria, were sent a reminder letter at three months after the original letter (*n* = 656), which yielded only a further 4 participants. Univariate linear regression models showed no evidence of an association between the number of children recruited by self-referral and practice list size (*p* = 0.092) or practice deprivation (*p* = 0.270).

**Table 1:** Reasons for general practitioner excluding children during screening of mail-out list for self-referral pathway

Reason for exclusion	Number excluded (%)
No longer has eczema	66 (37.3 %)
Not officially diagnosed	39 (22.0 %)
Parents/carers unable to complete questionnaires	23 (13.0 %)
Adverse medical or social circumstances	19 (10.7 %)
Other	30 (16.9 %)
Total	177 (100 %)

The six practices that were enlisted later to boost recruitment yielded 37.4 % of the recruitment by self-referral (40 of 107). Increasing the upper age limit to 5 years resulted in recruitment of 25 children aged between 3 and 5 years over 4 months, 17 by self-referral and 8 by in-consultation referral. This accounted for 12.6 % of total recruitment.

Recruitment yield from posters and leaflets in practice waiting rooms and word of mouth was small. Four children entered the study through these means, two of whom already had siblings in the study, one of whom was sign-posted into the study by a practice nurse, and one of whom saw a leaflet in the practice waiting room.

### Recruitment by in-consultation referral

Ninety (45.7 %) of 197 children were recruited by in-consultation referral. Univariate linear regression models showed no evidence of an association between the number of children recruited by practice through the in-consultation referral pathway and the practice ‘research level’ (*p* = 0.116) or the practice list size (*p* = 0.690). However, practices in a lower deprivation ‘decile’ (i.e., more deprived areas) were associated with higher numbers of children recruited in-consultation (*p* = 0.020).

Of the 56 practice staff given access to the randomisation database as requested by the practices to enable in-consultation referral, only slightly more than one-third (20 of 56 [35.7 %]) actually received consent (15 GPs, 1 pharmacist and 4 research nurses). Trial regulations meant that access to the randomisation database was gained via a personal identification number (PIN). Administering a unique PIN to each clinician was logistically challenging: PINs had to be sent in a password-protected email, which often had to be re-issued because of being misplaced. In addition, three incidences of children being randomised in error occurred. One when a clinician was familiarising themselves with the randomisation system, and two children who were randomised before their consent forms had been completed. These resulted in protocol breaches and generated additional work for the study team.

Relaxing regulatory restrictions to allow non-GCP-trained health professionals to receive consent (with the caveat that the consent form be countersigned by a person who was GCP-trained) resulted in recruitment of two participants by a non-GCP-trained clinician. The majority of consent was still taken by a GCP-trained professional.

**Fig. 3:**
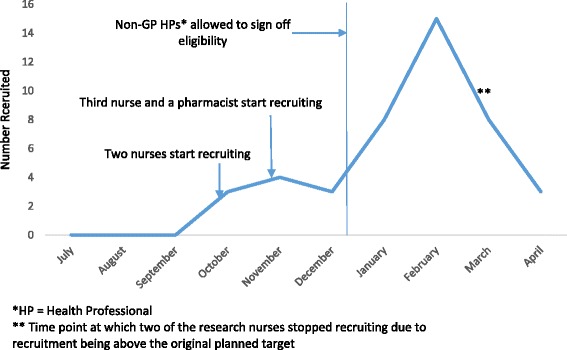
Recruitment per month by research nurses and pharmacist and implementation of recruitment strategy. *GP* General practitioner, *HP* Health professional

Figure 3 shows recruitment per month by the four research nurses and the pharmacist. The *arrow* indicates the time point at which they, instead of the GP, could confirm eligibility. There is a sharp increase in recruitment after this time point, with 12 children recruited in the five months prior to this change in protocol compared with 32 children in the latter four months. The drop in recruitment rate between March and April, as shown in Fig. 3, reflects the fact that two of the research nurses were asked to cease recruitment at the end of March because recruitment was above the original planned target at that stage. Overall, they recruited almost half of the children via this pathway (44 of 90 [48.9 %]). There was minimal difference in the withdrawal rate between those recruited by the research nurses and the pharmacist versus those recruited by the GPs (23.5 % versus 25 %, respectively).

Anecdotally, the study team found that this referral pathway generated additional work. Much of this was due to incomplete or incorrect paperwork by practice staff, including errors in consent form completion and missing case report form pages. Consequently, there were multiple communications back and forth with practices. Time was also spent visiting practices to promote more and better in-consultation recruitment (due to slower-than-expected rates). This additional effort appeared to have minimal impact because practices already recruiting well continued to do so, and activity in practices with little or no recruitment via this pathway did not increase their rates.

## Discussion

In the COMET feasibility trial, using two recruitment pathways and implementing additional strategies during the course of the study resulted in participant recruitment above the original planned target. Accrual was similar between the two recruitment pathways, with slightly fewer children recruited by in-consultation. This is contrary to what we had expected – that patients presenting to clinicians were more likely to be ‘emollient naive’ and therefore more willing to take part in the study. Interestingly, recruitment by this pathway was higher in practices in more deprived areas (*p* = 0.020). This probably reflects the fact that our highest recruiting clinicians were based in those practices, but it may warrant further exploration in the other studies. This pathway was more labour-intensive for the trial staff because of poorer-quality data collection by practice staff and a higher number of data queries. Withdrawal rates were also higher for the in-consultation pathway, which may reflect insufficient time or explanation of what was involved in taking part in the trial.

Our evaluation showed that a number of strategies worked in combination to increase cumulative recruitment. Several factors should be considered when recruiting children to trials in primary care: target population, recruiting via healthcare professionals and use of multiple recruitment strategies.

### Target population

‘No longer has eczema’ was the primary reason for exclusion at the screening stage prior to the mail-out (*n* = 66) and for parents declining participation (152 of 246 [61.8 %]). A large proportion of those identified for the mail-out were also excluded because they had not been diagnosed by a healthcare professional (*n* = 39). Thus, in similar populations where diagnosis may be uncertain or transient, such as patients with childhood asthma, it should be expected that a large proportion of potentially eligible patients will not have a confirmed diagnosis or will no longer have the condition, as the Read codes in electronic medical records cannot be relied upon to exclude such patients. Accounting for this should aid calculations of return and recruitment rates for trials that employ this method of recruitment. The specificity of searches for potentially eligible participants can probably be improved by adding search criteria such as evidence of active disease through a recent relevant prescription, as in the Bath Additives in the Treatment of Childhood Eczema (BATHE) study [31]. The decision regarding our original, narrower age range for eligibility was made on the basis that we thought older children were more likely to have an established treatment regimen and therefore would be less willing to be randomised to a different emollient. However, a recruitment yield of 12.6 % for children between the ages of 3 and 5 years (after the age criteria were broadened) suggests that there are still many families with older children who have not yet found a preferred emollient. Extrapolating from this result, we speculate that it is possible that, had the upper age limit been 5 years from the start of the study, an additional 26 % (taking an average of an additional six children per month) might have been recruited over the full 10-month recruitment period. Despite evidence derived from survey literature that reminders are helpful in increasing response rates [32], reminder invitation letters sent to non-responders in our trial yielded four participants. It may be that an interim of three months between the initial and reminder letters was too long. Telephone reminders may have been more generative, as indicated by the results of other primary care trials [33, 34]. The normal care pathway of the child and regulatory restrictions are also an important considerations. Many participants were seen and recruited by nurses, but governance restrictions on who could complete the trial paperwork hampered recruitment by this route. When these restrictions were relaxed, there was a rapid increase in the number of patients being referred into the trial by health professionals other than doctors (Fig. 3). When designing trials, researchers should give consideration to who normally diagnoses and treats the condition in question. If this includes health professionals other than doctors, they should be included in the eligibility criteria if at all possible.

### Recruiting via healthcare professionals

The number of recruiting clinicians was much less than expected, with only about one-third of clinicians able to randomise actually referring a patient into the study. Frank discussions with practices about the work involved in recruiting participants should mean that only health professionals who are able to fully commit to the study agree to be involved. We found that recruitment by health professionals was associated with more procedural problems and incorrectly completed paperwork, even though on-site training was provided, the clinician reimbursement costs were generous, and the paperwork was completed or countersigned by a GCP-trained professional. To ensure high-quality and consistent research processes for consent and data collection, researchers should not make any assumptions about the competence of GCP-trained clinicians. Ongoing support from the trial team was essential to keeping practice staff engaged with our trial. Continued support from the trial staff should be factored into the trial design in terms of time and financial support. Practice nurses at participating practices in our trial were better at recruiting, in terms of both recruitment yield and observing research processes. The literature is increasingly recognising the importance of the role of nurses in recruitment in primary care [10, 14, 35]. Researchers in future trials may wish to focus on nurses recruiting participants, depending on the population under investigation and their routine care pathway.

### Multiple recruitment strategies

We predicted that the in-consultation referral would work more favourably than the self-referral pathway, but both pathways produced very similar numbers of participants. The self-referral pathway was key in the early stages of recruitment because it took a while for the general practices to start recruiting by in-consultation. Self-referral also provided a steady influx of participants whom the study team had some control over by dictating the timing of the mail-outs, whereas the in-consultation route was more unpredictable. Therefore, we advise using a number of recruitment strategies to spread the risk, should one strategy prove more challenging or less fruitful than another. This is in agreement with other trial researchers who recommend using a number of strategies [36, 37]. We also emphasise the importance of careful recruitment monitoring because the additional measures we implemented during the trial (such as increasing the upper age limit and recruiting more practices) had an important and positive impact on recruitment and may not necessarily have been identified without monitoring.

### Limitations

First, there is a lack of evidence about the consistency with which the trial information was provided to parents/carers by clinicians versus the study team, with anecdotal evidence suggesting that self-referral participants were better informed than in-consultation participants about the trial and its commitments. Second, owing to poor completion of clinician screening logs, we have very limited data on the number of children approached in-consultation and reasons for non-participation. This information would have been useful for developing successful strategies. Third, we did not collect any ‘workload’ measures per pathway, because we did not anticipate this to be relevant, but, in hindsight, we could have tried to quantify this. Fourth, nested qualitative work might have helped us understand the issues better. Fifth, although this trial was classified as a CTIMP, it was a very low-risk trial using investigational medicinal products that can be bought over the counter, so some of the findings may not be applicable to higher-risk CTIMPs. This, and future feasibility studies, may benefit from a qualitative component to facilitate further understanding of feasibility issues.

## Conclusions

Shared learning of effective recruitment strategies is important for supporting the design of future trials to ensure successful recruitment, particularly for children in primary care trials. In our study, self-referral and in-consultation recruitment yielded similar numbers, though there were more barriers to efficient recruitment via the in-consultation pathway. Both pathways were supported by recruitment strategies implemented mid-study which worked to boost cumulative recruitment. Researchers in future trials of this type should consider the condition, care pathway and number of potentially eligible children and be prepared to use multiple recruitment strategies to achieve recruitment targets.

## Abbreviations

BATHE: Bath Additives in the Treatment of Childhood Eczema study
COMET: Choice of Moisturiser in Eczema Treatment trial
CONSORT: Consolidated Standards of Reporting Trials
CTIMP: Clinical trial of investigational medicinal products
RCT: Randomised controlled trial
GP: General practitioner
NRES: National Research Ethics Service
NOS: Not otherwise specified
ID: Identification number
SSC: NHS Service Support Costs
GCP: Good clinical practice
NIHR CRN: National Institute for Health Research Clinical Research Network
PIN: Personal identification number
PN: Practice nurse
